# From illusion to reality and back in time perception

**DOI:** 10.3389/fpsyg.2022.1031564

**Published:** 2022-10-10

**Authors:** Joseph Glicksohn

**Affiliations:** ^1^Department of Criminology, Bar-Ilan University, Ramat Gan, Israel; ^2^The Leslie and Susan Gonda (Goldschmied) Multidisciplinary Brain Research Center, Bar-Ilan University, Ramat Gan, Israel

**Keywords:** time perception, Gestalt psychology, flow of time, illusion, time estimation

Assuming that time perception is, indeed, a form of *perception* (Glicksohn, 2001)—an area acknowledged as such, now and again, in textbooks on perception (Murch, 1973; Chap. 7; Coren et al., 2004; Chap. 11)—one can address what Gruber et al. (2022) refer to as the “two times problem”, as a problem for perception, and not one whose resolution must necessarily span between psychology and physics. Indeed, in agreement with Smythies (2003, p. 53), who suggests that “if one wants to account for our psychological impression that there is a ‘now’ in time and moreover that time in some way flows, we must look elsewhere than contemporary physics, whether Newtonian or Relativity, to find it”, it would be instructive to return to the Gestalt psychologist Kurt Koffka, who posed the classic question for theorists of perception, namely “why do things look as they do?” (Koffka, 1935; p. 76). In the present context, this can be rephrased as “why do we perceive time the way we do?” While Gruber et al. (2022) draw a distinction between “the veridical and illusory nature of time”, for the Gestalt psychologists, as Epstein and Hatfield (1994, p. 166) stress, “phenomenal experience is real … it is not illusory or suspect in any way.” Hence, even if the *flow* of time is considered to be illusory (Gruber et al., 2015), while time *estimation* might well be “real” (Gruber et al., 2020), both need to be addressed by psychology.

In a recent paper (Glicksohn and Ben-Soussan, under review)^1^, it has been suggested that “While a minority of researchers … accepted that subjective time could be neither veridical nor linear… the majority embraced … [the] view that subjective time could be both veridical and linear.” Either way, for Gruber et al. (2022) this would imply that subjective time (or, *apparent duration, psychological time*, or *estimated time*; Glicksohn, 2001; Buhusi and Meck, 2009) would be “real” (veridical, or not), to be contrasted with the flow of time (or, *temporal flow, passage of time judgment*, or *perceived speed of time*; Larson and von Eye, 2006; Wearden, 2015; Droit-Volet, 2018; Thönes et al., 2018; Vogel et al., 2020; Martinelli and Droit-Volet, 2022), which is “illusory”. And yet illusions (flow of time?), as Zavagno et al. (2015) have argued, “can be effective tools in studying the brain in reference to perception and also to cognition in a much broader sense.” Hence, even if the flow of time is an illusory construct, it might still be either correlated with subjective time (Eisler and Eisler, 2009) or dissociated from this (Wittmann et al., 2015; Droit-Volet and Wearden, 2016; Hancock et al., 2019). Of particular significance is the fact that the flow of time can be indicative of a state of flow (Larson and von Eye, 2006; Hancock et al., 2019; Kent et al., 2022) or a state of absorption (Woodrow, 1951; Glicksohn and Lipperman-Kreda, 2007; Glicksohn and Berkovich-Ohana, 2012; Mohr, 2018) in an ongoing activity.

Maybe, however, the flow of time is not an illusory construct. Koffka would probably disagree with Gibson's (1979) answer to his question, “that things look as they do because the information in proximal stimulation is what it is” (Epstein, 1994; p. 176).In turn, Gibson (1975) himself would probably argue against the very notion of time *perception*, and that time itself “is ‘real’ and can be directly perceived” (Larson and von Eye, 2006, p. 114). Nevertheless, it is still the case that the optic flow (Rogers, 2021) with which Gibson was primarily concerned might well be a ready source for the flow of time. Consider the case when your plane is descending toward the airport, and from the vantage of your window the optic flow is in continual flux. If the velocity were constant, your perceptual experience would be very different from the usual case wherein the plane is decelerating. One would not be surprised if the corresponding flow of time was also altered. In a recent study, a group of researchers looked at the flow of time on exposure to a starfield environment, and reported that “Passage of time experience was increased for faster stars and more dense starfields, but was not as much affected by the actual duration of the interval or the task difficulty. This shows that the salience of moment-to moment differences between individual frames is more directly associated with the experience of passage of time than is the actual duration of the interval” (Jording et al., 2022; p. 12). This comes in support of the suggestion made here that changes in optic flow might very well affect the flow of time. If that were the case, would both be considered to be illusory?

A reviewer of this commentary has questioned whether the issue of temporal *continuity* is ever *directly addressed* in passage of time judgments. Gruber et al. (2022) refer to the question asked of observers of “how fast time went”—namely, whether time was felt to pass quickly or slowly. As the reviewer astutely notes, that type of judgment can be affected by such a factor as boredom. Hence, the less or more bored one feels will affect the subsequent change in passage of time judgment, irrespective of the impact of the change in optic flow (as suggested here). My suggestion would be, therefore, to employ a question referring to the present, ongoing, subjective experience of the flow of time. For example, in a study employing virtual reality (Glicksohn and Avnon, 1997–1998), the experimenter lightly tapped the shoulder of the participant during the session, signaling the request for an introspective report. In a similar manner, one could send a text message to the participant asking for a current rating of the subjective experience of the flow of time. While this is certainly feasible, one should also consider the fact that in doing so, one is actually momentarily disrupting the ongoing experience of that participant. Sometimes, this can be fatal for the subjective experience under investigation. Nonetheless, in order to make a stronger argument regarding the suggestion made here relating change in optic flow with change in reported passage of time, this would be a necessary requirement for a future study in this domain.

A second way in which the flow of time might be affected can be derived from the multiplicative model for apparent duration (Glicksohn, 2001). According to this model, time production is a multiplicative function of two components: The size of the subjective time unit (which varies with context), and the number of these subjective time units. Kent et al. (2019) have recently applied this model in their discussion of time dilation, especially that related to depression. They suggest that “the mode of prospective time judgment in production tasks changes as intervals increase from around 1 s of the experienced moment into the 30 s range of mental presence” (p. 80). Specifically, “if it is assumed that the size of Glicksohn's (2001) time units can vary within the same interval, then units at the end of the interval will be *relatively* small compared to intervals at the beginning of the interval” (p. 78). This would suggest that “time accelerates as intervals increase, an effect which in itself may not be unique to depression. It may be a general feature of time perception that is simply more pronounced for depressed individuals” (p. 78). Hence, a reported change in flow of time might well be related to a discontinuity in time-production data [Glicksohn et al., 2017; Glicksohn and Ben-Soussan, under review (see text footnote 1)]. Indeed, as Martinelli and Droit-Volet (2022, p. 528) have recently suggested, the passage of time judgment curve “might not be as linear as observed” in their study, given extreme conditions. Perhaps, as Gruber et al. (2022, p. 1) argue, “the veridical system is a reflection of accepted spacetime cosmologies and through natural selection begets the illusory system for functional purposes”. What this means for the “two times problem” discussed by Gruber et al. (2022) is that one needs to consider not only the question of temporal continuity, which they believe to be an illusory experience, but also that of temporal *discontinuity*. It is not, however, clear to me whether such temporal discontinuity would also be considered to be an illusory experience.

Perhaps, as Conway et al. (2016) suggest, “humans should have a psychological mechanism for slowing time down as motion speeds up”—what they refer to as a “spacetime processor”. Who knows? Gruber et al. (2022) have certainly given us plenty of food for thought.

## Author contributions

The author confirms being the sole contributor of this work and has approved it for publication.

## Conflict of interest

The author declares that the research was conducted in the absence of any commercial or financial relationships that could be construed as a potential conflict of interest.

## Publisher's note

All claims expressed in this article are solely those of the authors and do not necessarily represent those of their affiliated organizations, or those of the publisher, the editors and the reviewers. Any product that may be evaluated in this article, or claim that may be made by its manufacturer, is not guaranteed or endorsed by the publisher.

## Footnotes

^1^Glicksohn, J., and Ben-Soussan, T. D. (under review). Discontinuity in time perception, or inadequacy of psychophysical fit?

## References

[B1] Buhusi C. V. Meck W. H. (2009). Relativity theory and time perception: single or multiple clocks? PLoS ONE 4, e6268. 10.1371/journal.pone.0006268 19623247 PMC2707607

[B2] Conway L. G. Repke M. A. Houck S. C. (2016). Psychological spacetime: Implications of relativity theory for time perception. SAGE Open 6, 1–14. 10.1177/2158244016674511

[B3] Coren S. Ward L. M. Enns J. T. (2004). Sensation and perception. Hoboken, NJ: John Wiley and Sons.

[B4] Droit-Volet S. (2018). Intertwined facets of subjective time. Curr. Direct. Psychol. Sci. 27, 422–428. 10.1177/0963721418779978

[B5] Droit-Volet S. Wearden J. (2016). Passage of time judgments are not duration judgments: evidence from a study using experience sampling methodology. Front. Psychol. 7, 176. 10.3389/fpsyg.2016.00176 26925006 PMC4759643

[B6] Eisler A. D. Eisler H. (2009). Experienced speed of time in durations of known and unknown length. NeuroQuantology 7, 66–76. 10.14704/nq.2009.7.1.208

[B7] Epstein W. (1994). “Why do things look as they do?”: What Koffka might have said to Gibson, Marr and Rock,” in Gestalt Psychology: Its Origins, Foundations and Influence, ed S. Poggi (Firenze: Leo S. Oschki Editore), 175–189.

[B8] Epstein W. Hatfield G. (1994). Gestalt psychology and the philosophy of mind. Philos. Psychol. 7, 163–181. 10.1080/09515089408573118

[B9] Gibson J. J. (1975). “Events are perceivable but time is not,” in The Study of Time II, eds J. T. Fraser, and N. Lawrence (New York: Springer-Verlag), 295–301.

[B10] Gibson J. J. (1979). The Ecological Approach to Visual Perception. Boston: Houghton Mifflin.

[B11] Glicksohn J. (2001). Temporal cognition and the phenomenology of time: a multiplicative function for apparent duration. Consc. Cognit. 10, 1–25. 10.1006/ccog.2000.0468 11273623

[B12] Glicksohn J. Avnon M. (1997–1998). Explorations in virtual reality: absorption, cognition and altered state of consciousness. Imag. Cognit. Person. 17, 141–151. 10.2190/FTUU-GLC5-GBT8-9RUW

[B13] Glicksohn J. Berkovich-Ohana A. (2012). “Absorption, immersion, and consciousness,” in Video Game Play and Consciousness, ed J. Gackenbach (New York: Nova Science Publishers, Inc.), 83–99.

[B14] Glicksohn J. Berkovich-Ohana A. Mauro F. Ben-Soussan T. D. (2017). Time perception and the experience of time when immersed in an altered sensory environment. Front. Hum. Neurosci. 11, 487. 10.3389/fnhum.2017.00487 29056902 PMC5635043

[B15] Glicksohn J. Lipperman-Kreda S. (2007). Time, thought, and consciousness. J. Mind Behav. 28, 289–305.

[B16] Gruber R. P. Bach M. Block R. A. (2015). Perceiving two levels of the flow of time. J. Consc. Stud. 22, 7–22.

[B17] Gruber R. P. Block R. A. Montemayor C. (2022). Physical time within human time. Front. Psychol. 13, 718505. 10.3389/fpsyg.2022.718505 35432085 PMC9005802

[B18] Gruber R. P. Montemayor C. Block R. A. (2020). From physical time to a dualistic model of human time. Found. Sci. 25, 927–954. 10.1007/s10699-020-09670-4

[B19] Hancock P. A. Kaplan A. D. Cruit J. K. Hancock G. M. MacArthur K. R. Szalma J. L. (2019). A meta-analysis of flow effects and the perception of time. Acta Psychol. 198, 102836. 10.1016/j.actpsy.2019.04.007 31279183

[B20] Jording M. Vogel D. H. Viswanathan S. Vogeley K. (2022). Dissociating passage and duration of time experiences through the intensity of ongoing visual change. Sci. Rep. 12, 1–15. 10.1038/s41598-022-12063-1 35581249 PMC9113985

[B21] Kent L. Nelson B. Northoff G. (2022). Can disorders of subjective time inform the differential diagnosis of psychiatric disorders? A transdiagnostic taxonomy of time. Early Intervent. Psychiatry. 10.1111/eip.13333 36935204

[B22] Kent L. Van Doorn G. Klein B. (2019). Time dilation and acceleration in depression. Acta Psychol. 194, 77–86. 10.1016/j.actpsy.2019.02.003 30798221

[B23] Koffka K. (1935). Principles of Gestalt Psychology. New York, NY: Harcourt, Brace and World.

[B24] Larson E. von Eye A. (2006). Predicting the perceived flow of time from qualities of activity and depth of engagement. Ecol. Psychol. 18, 113–130. 10.1207/s15326969eco1802_3

[B25] Martinelli N. Droit-Volet S. (2022). What factors underlie our experience of the passage of time? Theoretical consequences. Psychol. Res. 86, 522–530. 10.1007/s00426-021-01486-6 33760972

[B26] Mohr C. (2018). Are there varying depths in flow? Altered states of consciousness, absorption, and the brain. J. Consc. Stud. 25, 115–130.

[B27] Murch G. M. (1973). Visual and Auditory Perception. New York, NY: The Bobbs-Merrill Company.

[B28] Rogers B. (2021). Optic flow: perceiving and acting in a 3-D world. i-Perception 12, 1–25. 10.1177/2041669520987257 33613957 PMC7869175

[B29] Smythies J. (2003). Space, time and consciousness. J. Consc. Stud. 10, 47–56.

[B30] Thönes S. Arnau S. Wascher E. (2018). Cognitions about time affect perception, behavior, and physiology: a review on effects of external clock-speed manipulations. Consc. Cognit. 63, 99–109. 10.1016/j.concog.2018.06.014 29966862

[B31] Vogel D. H. Jording M. Kupke C. Vogeley K. (2020). The temporality of situated cognition. Front. Psychol. 11, 546212. 10.3389/fpsyg.2020.546212 33132954 PMC7550648

[B32] Wearden J. H. (2015). Passage of time judgements. Consc. Cognit. 38, 165–171. 10.1016/j.concog.2015.06.005 26115594

[B33] Wittmann M. Otten S. Schötz E. Sarikaya A. Lehnen H. Jo H.-G.. (2015). Subjective expansion of extended time-spans in experienced meditators. Front. Psychol. 5, 1586. 10.3389/fpsyg.2014.01586 25642205 PMC4294119

[B34] Woodrow H. (1951). “Time perception,” in Handbook of Experimental Psychology, ed S. S. Stevens (New York, NY: Wiley), 1224–1236.

[B35] Zavagno D. Daneyko O. Actis-Grosso R. (2015). Mishaps, errors, and cognitive experiences: on the conceptualization of perceptual illusions. Front. Hum. Neurosci. 9, 190. 10.3389/fnhum.2015.00190 25918504 PMC4394699

